# Arsenite Impairs BRCA1-Dependent DNA Double-Strand Break Repair, a Mechanism Potentially Contributing to Genomic Instability

**DOI:** 10.3390/ijms241814395

**Published:** 2023-09-21

**Authors:** Tizia Matthäus, Sandra Stößer, Hatice Yasemin Seren, Vivien M. M. Haberland, Andrea Hartwig

**Affiliations:** Department of Food Chemistry and Toxicology, Institute of Applied Biosciences (IAB), Karlsruhe Institute of Technology (KIT), Adenauerring 20a, 76131 Karlsruhe, Germany

**Keywords:** arsenite, DSB repair, BRCA1, HR, NHEJ, gene expression, RAD51, RAD54, 53BP1, DNA-PKcs, apoptosis

## Abstract

BRCA1 is a key player in maintaining genomic integrity with multiple functions in DNA damage response (DDR) mechanisms. Due to its thiol-rich zinc-complexing domain, the protein may also be a potential target for redox-active and/or thiol-reactive (semi)metal compounds. The latter includes trivalent inorganic arsenic, which is indirectly genotoxic via induction of oxidative stress and inhibition of DNA repair pathways. In the present study, we investigated the effect of NaAsO_2_ on the transcriptional and functional DDR. Particular attention was paid to the potential impairment of BRCA1-mediated DDR mechanisms by arsenite by comparing BRCA1-deficient and -proficient cells. At the transcriptional level, arsenite itself activated several DDR mechanisms, including a pronounced oxidative stress and DNA damage response, mostly independent of BRCA1 status. However, at the functional level, a clear BRCA1 dependency was observed in both cell cycle regulation and cell death mechanisms after arsenite exposure. Furthermore, in the absence of arsenite, the lack of functional BRCA1 impaired the largely error-free homologous recombination (HR), leading to a shift towards the error-prone non-homologous end-joining (NHEJ). Arsenic treatment also induced this shift in BRCA1-proficient cells, indicating BRCA1 inactivation. Although BRCA1 bound to DNA DSBs induced via ionizing radiation, its dissociation was impaired, similarly to the downstream proteins RAD51 and RAD54. A shift from HR to NHEJ by arsenite was further supported by corresponding reporter gene assays. Taken together, arsenite appears to negatively affect HR via functional inactivation of BRCA1, possibly by interacting with its RING finger structure, which may compromise genomic stability.

## 1. Introduction

DNA is the carrier of an organism’s genetic information and is under constant attack from exogenous and endogenous factors. Since the survival of an organism depends on the transmission of genetic information to its subsequent generations, the cell possesses a complex network of different mechanisms to maintain genomic stability [1]. The BRCA1 (breast cancer type 1 susceptibility protein) is a core component of this signal transduction cascade, known as the DNA damage response (DDR). BRCA1 has previously been shown to interact with several signaling proteins following exposure to ionizing radiation (IR) [2,3]. The importance of BRCA1 is particularly evident in studies on *BRCA1*-mutant cells; a loss-of-function mutation increases the likelihood of tumor formation, particularly breast and ovarian cancer [4,5]. Therefore, one goal of this study was to gain more insight into the impact of BRCA1 on the different signaling pathways of the DDR.

From a toxicological point of view, BRCA1 is of particular interest because it contains a RING finger structure [3,4], which may represent a potentially sensitive target for redox-active thiol-affine metal and metalloid compounds. Thus, thiol groups within zinc finger structures in various proteins have previously been shown to be perturbed by toxic and/or carcinogenic metal compounds. The binding of metal ions to these critical thiol groups, together with oxidation, can lead to conformational changes and consequent loss of protein function [6]. However, up to now, there is little evidence that toxic metal compounds interfere with RING finger structures.

One such semi-metallic species that can potentially disrupt zinc-binding structures is trivalent inorganic arsenic. Worldwide, several million people have been exposed to arsenite through drinking water or food, and increased intake correlates with a higher risk of lung, skin, and bladder cancer. Consequently, arsenic and its inorganic compounds have been classified as human carcinogens (Group 1) by the International Agency for Research on Cancer (IARC) and the German MAK Commission [7,8]. While direct mutagenicity of arsenite appears to be of minor importance, indirect genotoxicity, including the induction of oxidative stress and the interactions with DNA repair processes, has been demonstrated in several studies (reviewed in [9]).

An underlying molecular mechanism of arsenite is based on its strong affinity for adjacent dithiol and trithiol groups, which are present in numerous zinc-binding domains of proteins, including many transcription factors as well as DNA repair and tumor suppressor proteins [6]. The impairment of zinc finger proteins by arsenite has been demonstrated previously in several studies [10,11,12,13], with poly(ADP-ribose)polymerase 1 (PARP-1) being one particularly sensitive target [14]. In contrast, little is known about arsenite-mediated interference with RING finger proteins. Initial evidence was provided by the study of Zhang et al., who described arsenite binding to the RING domains of an E3 ubiquitin ligase with impaired H2B ubiquitination and impaired DNA double-strand break repair (DSBR). Furthermore, they described reduced binding of BRCA1 and RAD51 [15].

By comparing BRCA1-proficient and -deficient cell lines, the present study aimed to investigate the impact of arsenite on BRCA1 expression and function in DNA DSB repair. We found that arsenite clearly activated the transcriptional oxidative stress and DNA damage responses. The absence of a functional BRCA1 protein impaired arsenite-induced cell cycle arrest; furthermore, the function of BRCA1 protein in DNA DSB repair and downstream protein binding was disrupted by arsenite, with adverse consequences, especially for HR.

## 2. Results

### 2.1. Cytotoxicity of Arsenite

**Figure 1:**
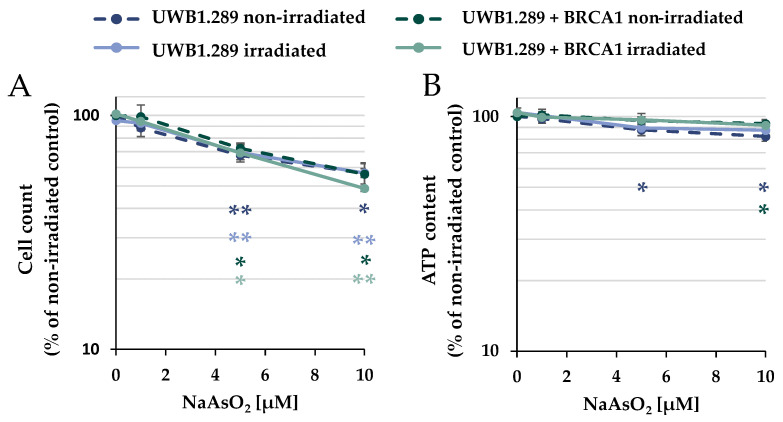
Cytotoxicity of arsenite in UWB1.289 and UWB1.289 + BRCA1 cells. Cell count (**A**) and ATP content (**B**) were determined after treatment with NaAsO_2_. Cells were pre-incubated with NaAsO_2_ for 18 h, irradiated with 1 Gy, or left unirradiated and post-incubated with NaAsO_2_ for 8 h. For the non-irradiated cells, this resulted in a total incubation time of 26 h. Shown are mean values ± standard deviations derived from three independent experiments. Statistically significant difference from the control of the respective cell line as determined using ANOVA followed by Dunnett’s T3 post hoc test: * *p* ≤ 0.05, ** *p* ≤ 0.01.

The cytotoxic potential of arsenite was evaluated after treatment with NaAsO_2_ in the presence or absence of IR on UWB1.289 (BRCA1-null ovarian cancer cell line; BRCA1-deficient) and UWB1.289 + BRCA1 (BRCA1-wild-type, BRCA1-proficient) cells to define appropriate incubation conditions. To assess viability, the cellular ATP content was determined after NaAsO_2_ treatment, in addition to the cell number. The cells were pre-incubated with NaAsO_2_ for 18 h, irradiated with 1 Gy, or left unirradiated, and post-incubated with NaAsO_2_ for 8 h, resulting in a total incubation time with arsenite of 26 h (Figure 1). In terms of cell number, a concentration-dependent and similar cytotoxic potential of NaAsO_2_ was observed in both cell lines, starting at 5 µM; irradiation had no further effect on cytotoxicity. Similarly, ionizing radiation had no effect on the ATP content, and arsenite alone as well as after co-treatment with irradiation only marginally affected this parameter. Thus, the two methods identified mild to moderate cytotoxic effects by treatment with 5 µM and moderate to strong cytotoxic effects by 10 µM NaAsO_2_ in both UWB1.289 and UWB1.289 + BRCA1 cells. Therefore, the following experiments were performed with the same incubation conditions at arsenite concentrations of up to 10 µM, except for immunofluorescence studies, if not stated otherwise.

### 2.2. Uptake and Intracellular Accumulation

**Figure 2:**
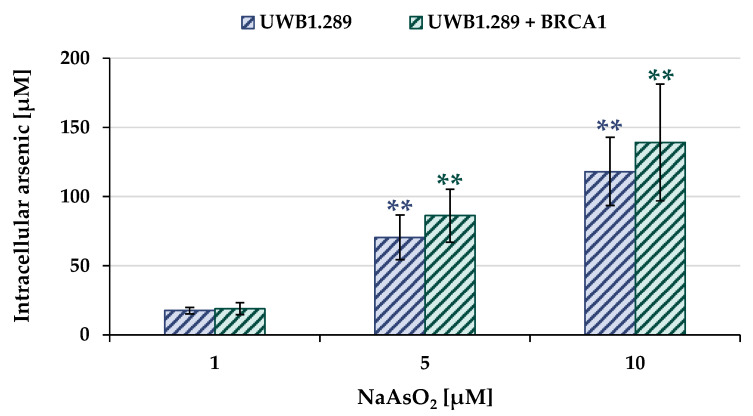
Accumulation of arsenic in UWB1.289 and UWB1.289 + BRCA1 cells following treatment with NaAsO_2_ for 18 h. Shown are mean values ± standard deviations derived from three independent experiments. Statistically significant difference from untreated control of the respective cell line as determined by paired, two-tailed student’s *t*-test: ** *p* ≤ 0.01.

To determine the arsenic uptake as a function of extracellular arsenite concentration, total arsenic concentration was measured by atomic absorption spectroscopy (AAS). The UWB1.289 and UWB1.289 + BRCA1 cells were incubated with NaAsO_2_ for 18 h, and the results for both cell lines are shown in Figure 2.

Arsenite treatment resulted in a concentration-dependent and pronounced accumulation of intracellular arsenic in both BRCA1-proficient and BRCA1-deficient cells. In the case of 1 µM extracellular arsenite, 20 µM intracellular arsenic was found, and incubation with 10 µM NaAsO_2_ resulted in intracellular arsenic levels ranging from 120 µM to 140 µM. No statistically significant differences were found between the two cell lines, suggesting comparable arsenic uptake.

### 2.3. Gene Expression Profiling

**Figure 3:**
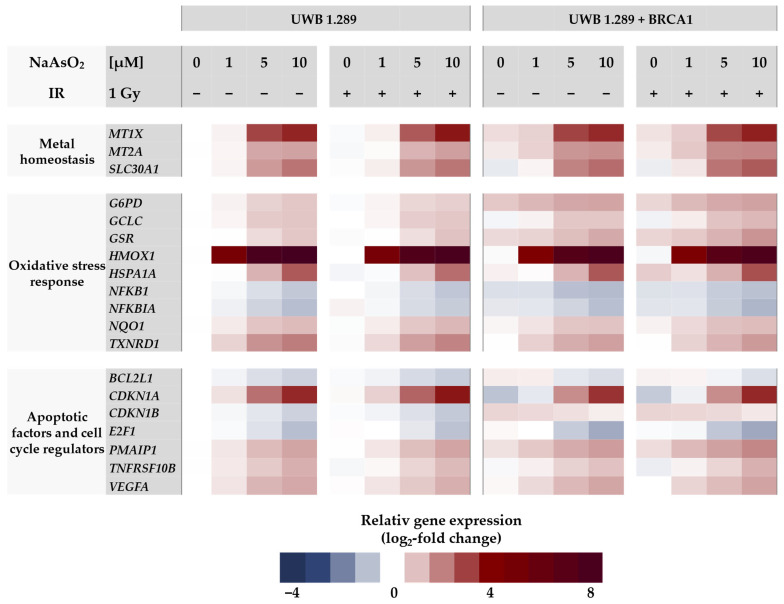
Gene expression profiling of UWB1.289 and UWB1.289 + BRCA1 cells after treatment with NaAsO_2_. Cells were pre-incubated with NaAsO_2_ for 18 h, irradiated with 1 Gy, or left unirradiated and post-incubated with NaAsO_2_ for 8 h. This resulted in a total incubation time with arsenite of 26 h. Gene expression was determined using high-throughput RT-qPCR. Genes were classified into the clusters of metal homeostasis, oxidative stress response, apoptotic factors, and cell cycle regulators as well as DNA damage response. The log_2_-fold changes are derived as mean values from three independent experiments, normalized to the untreated control of the respective cell line, with the control equal to 0.

**Figure 4:**
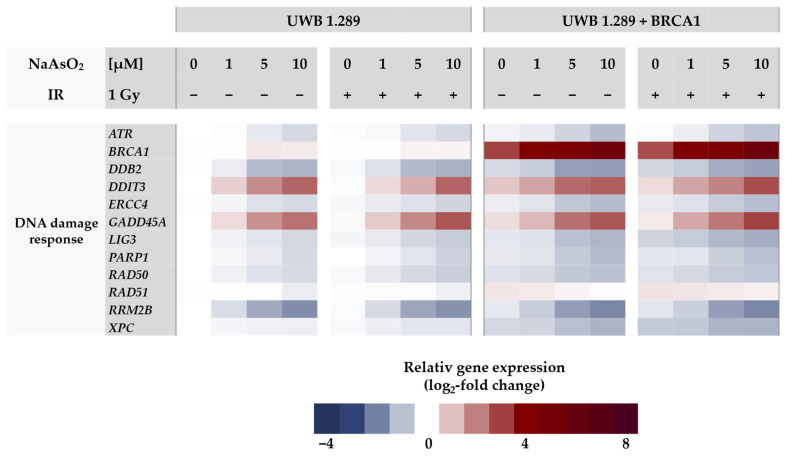
Gene expression profiling of UWB1.289 and UWB1.289 + BRCA1 cells after treatment with NaAsO_2_. Cells were pre-incubated with NaAsO_2_ for 18 h, irradiated with 1 Gy, or left unirradiated and post-incubated with NaAsO_2_ for 8 h. For the non-irradiated cells, this resulted in a total incubation time of 26 h. Gene expression was determined using high-throughput RT-qPCR. Genes were classified into the clusters of metal homeostasis, oxidative stress response, apoptotic factors, and cell cycle regulators as well as DNA damage response. The log_2_-fold changes are derived as mean values from three independent experiments, normalized to the untreated control of the respective cell line, with control equal to 0.

In the next step, gene expression profiles were generated to identify the cellular response to arsenite in BRCA1-proficient cells compared to BRCA1-deficient cells. For this purpose, a high-throughput RT-qPCR was used, which allowed the parallel analysis of 96 samples on the expression of 95 genes related to genomic stability. With this approach, native BRCA1-deficient UWB1.289 and transfected BRCA1-proficient UWB1.289 + BRCA1 cells were compared. The gene set included markers of metal homeostasis, (oxidative) stress response, DNA damage and repair, cell cycle control, and apoptosis. A detailed description, including a complete list of the respective primers, genes, and their encoded proteins has been published previously by our group [16]. Relative gene expression was calculated by normalizing the treated samples to the untreated control of each cell line and are expressed as log_2_ values. A reduction of at least 50% (log_2_-fold change ≤ −1) or a doubling (log_2_-fold change ≥ 1) compared to the untreated control was considered relevant, but concentration-dependent trends were also considered. The gene expression profiles for both cell lines are shown as heat maps in Figure 3 and Figure 4. To assess the effect of NaAsO_2_, the untreated control of the respective cell line was chosen as a reference. Comparing the gene expression profiles of the two cell lines under basal conditions, only the transcription rate of the *BRCA1* gene showed a respective difference, confirming the BRCA1 deficiency and BRCA1 proficiency, respectively. Regarding NaAsO_2_ treatment, both cell lines showed a similar gene expression pattern. Neither ionizing radiation alone nor after co-treatment with NaAsO_2_ showed any difference in its effect on transcriptional activity.

As shown in Figure 3, no changes at the transcriptional level were observed after ionizing radiation, indicating that no DDR was activated at this rather low level of irradiation. In contrast, a clear and dose-dependent effect was detected in the case of arsenite treatment. Here, the most pronounced changes were observed in the oxidative stress response upon treatment with arsenite. The greatest effect was observed for *HMOX1*, which encodes heme oxygenase 1; remarkably, a 5-fold induction was already observed at 1 µM arsenite when the lowest concentration was applied. Another sensor gene for oxidative stress is *HSPA1A*, whose transcript levels were also increased, although less pronounced and only detected at 5 µM arsenite. Rather small but dose-dependent inductions were observed for other oxidant-responsive genes such as *G6PD*, *GCLC,* and *GSR*, as well as for *NQO1* and *TXNRD1*. In contrast, slight repressions were observed for the genes *NFKB1* and *NFKBIA*. Among the metal homeostasis genes, *MT1X*, *MT2A*, encoding metallothioneins, and *SLC30A1*, encoding a zinc exporter from cells, showed a dose-dependent up-regulation after arsenite exposure, starting at 5 µM (2.5-fold induction).

Within the cluster of apoptotic factors and cell cycle regulators, the gene *CDKN1A*, which encodes the protein p21, showed a 2.5-fold increased expression at 5 µM arsenite. In addition, the expression of *PMAIP*, which encodes the pro-apoptotic BCL-2 protein Noxa, was 1.4-fold increased. The gene *TNFRSF10B*, encoding the death receptor DR5, also showed increased transcription. In contrast, arsenite induced repression of the proliferation-associated gene *E2F1* and the anti-apoptotic gene *BCL2L1*.

Regarding the DNA damage response gene cluster (Figure 4), arsenite activated genes related to DNA damage signaling, namely *GADD45A* and *DDIT3*, starting at 1 µM, as well as *BRCA1* in the proficient cell line. Interestingly, DNA damage signaling by *GADD45A* and *DDIT3* was more pronounced in the BRCA1-deficient cell line, indicating a lack of protection by *BRCA1* induction. In contrast, for all other genes encoding DNA repair factors such as *ATM, ATR, and PARP1* as well as those encoding specific DNA repair factors of all major DNA repair pathways (*DDB2, ERCC4, LIG1, LIG3, PARP1, PCNA, POLD1, RAD50, and XPC*), expression was down-regulated by arsenite in a dose-dependent manner, reaching levels between 50% and 30% of control. The repression was most pronounced in the case of *RRM2B* (ribonucleotide reductase regulatory TP53 inducible subunit M2B), which is required for DNA synthesis.

### 2.4. Cell Cycle Regulation

NaAsO_2_ treatment affected the expression of genes associated with cell cycle regulation and proliferation. To verify the results at the functional level, the cell cycle distribution in UWB1.289 and UWB1.289 + BRCA1 cells was subsequently analyzed using flow cytometry.

**Figure 5:**
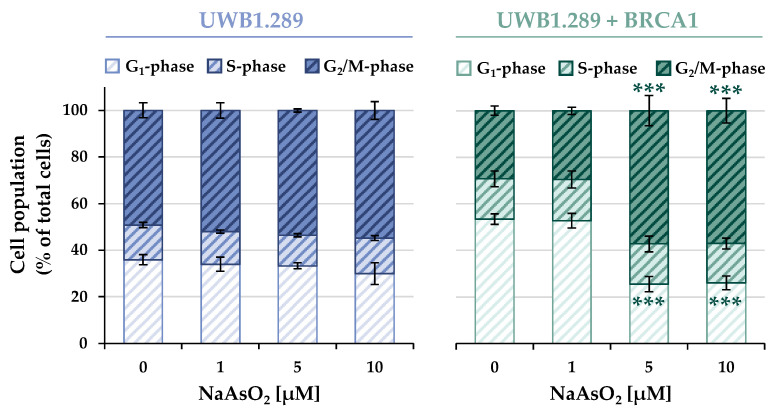
Analysis of the cell cycle distribution of UWB1.289 and UWB1.289 + BRCA1 cells after treatment with NaAsO_2_ for 26 h. Cell cycle distribution was analyzed by DAPI staining using flow cytometry. Shown are mean values ± standard deviations derived from three independent experiments. Statistically significant difference from control as determined using ANOVA followed by Dunnett’s T post hoc test: *** *p* ≤ 0.001.

The cell cycle distribution of the non-irradiated samples of both cell lines is shown in Figure 5, whereas the results of the irradiated cells are shown in Figure S1. In addition, a time-dependent analysis was performed, including 24, 48, 72, and 96 h post-treatment to determine the course of the cell cycle distribution and progression over time (Figures S2–S4).

In contrast to the results of the previous experiments, a clear difference was observed between the BRCA1-deficient and the BRCA1-proficient cells. Both the basal and the arsenite-induced cell cycle distributions showed distinct patterns in the two cell lines, with no further effect of ionizing radiation. Considering the basal cell cycle distribution (0 μM), in the BRCA1-deficient cells, the majority of the cell population was found in the G_2_/M phase (~50%), about 35% of the cells remained in the G1 phase, whereas the S phase (~17%) accounted for the smallest fraction of cells. In contrast, in BRCA1-proficient cells, the largest fraction of cells was found in the G_1_ phase (~53%), approximately 17% were in the S phase, and 30% of the cells were detected in the G_2_/M phase.

Arsenite treatment also revealed distinct differences between the two cell lines. While the BRCA1-deficient UWB1.289 cells largely maintained the basal phase distribution over the entire NaAsO_2_ concentration range, a clear G_2_/M arrest (~57%) was observed in the UWB1.289 + BRCA1 cells starting at the slightly cytotoxic level of 5 μM. This was associated with a decrease in the fraction of cells in the G_1_ phase (~26%), whereas the S phase cell population remained largely unchanged (~17%).

### 2.5. DNA Double-Strand Break Repair

Since the transcriptional DNA damage response including the expression of DNA repair factors after arsenite treatment was largely independent of the BRCA1 status of the cells, this was aimed to be verified at the functional level. IF staining was used to analyze the recruitment and dissociation of specific DNA repair proteins to the sites of DNA damage. To investigate repair kinetics, DNA double-strand breaks (DSB) were induced by 1 Gy irradiation, and proteins involved in homologous recombination (HR) and non-homologous end-joining (NHEJ) were analyzed. Cells were differentiated between G_1_ and G_2_ phases using flow cytometry, using centromere protein F (CENP-F) staining. Only cells in the G_2_ phase were analyzed, as both repair pathways are active in this phase of the cell cycle [17].

Cells were pre-incubated with arsenite for 18 h, irradiated with 1 Gy, or left unirradiated and post-incubated with arsenite for 1–24 h. In the case of the non-irradiated cells, this resulted in a total incubation time of 19–42 h. In contrast to the previous studies, only 1 μM as well as 5 μM NaAsO_2_ were applied, considering the sensitivity of this method. IF staining of the repair proteins mentioned below showed that arsenite treatment alone did not induce DNA DSBs at the concentrations used; therefore, the foci counts of the non-irradiated samples were subtracted from those of the irradiated batch in each case.

**Figure 6:**
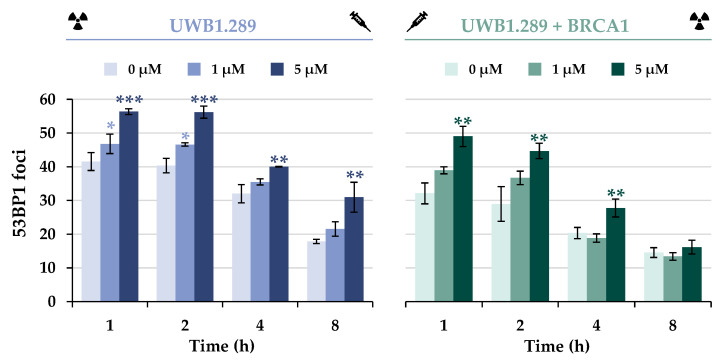
Immunostaining of 53BP1 in UWB1.289 and UWB1.289 + BRCA1 cells after treatment with NaAsO_2_. Cells were pre-incubated with NaAsO_2_ for 18 h, irradiated with 1 Gy, and post-incubated with NaAsO_2_ for 1 h to 8 h. Cells were stained against 53BP1 and manually counted. For each time point and treatment, foci in 40 cells of the G_2_ phase were counted, and the foci counts of the non-irradiated samples were subtracted from those of the irradiated batch. Shown are mean values ± standard deviations derived from three independent experiments. Statistically significant difference from control as determined using ANOVA followed by Dunnett’s T post hoc test: * *p* ≤ 0.05, ** *p* ≤ 0.01; *** *p* ≤ 0.001.

The initial repair of DSBs was monitored using the protein 53BP1, which is an essential factor in DSB damage recognition. At the molecular level, the equilibrium between 53BP1 and BRCA1 also plays a critical role in the selection of the DSB repair pathway by modulating end resection [18,19]. The results indicate recruitment of the DNA repair protein 53BP1 to IR-induced DSBs in the G_2_ phase of both cell lines (Figure 6 and Figure S5). A decrease in IR-generated foci to approximately 50% was observed with increasing time in both cell lines, suggesting dissociation with similar capacity.

Additional treatment with NaAsO_2_ had a synergistic effect in UWB1.289 and also in UWB1.289 + BRCA1 cells, indicating an accumulation of DNA DSBs at all time points examined, presumably due to decreased repair. However, a decrease in 53BP1 foci over time was also observed in co-exposed cells. Overall, fewer foci were detected in BRCA1-proficient cells, with a greater reduction over time.

**Figure 7:**
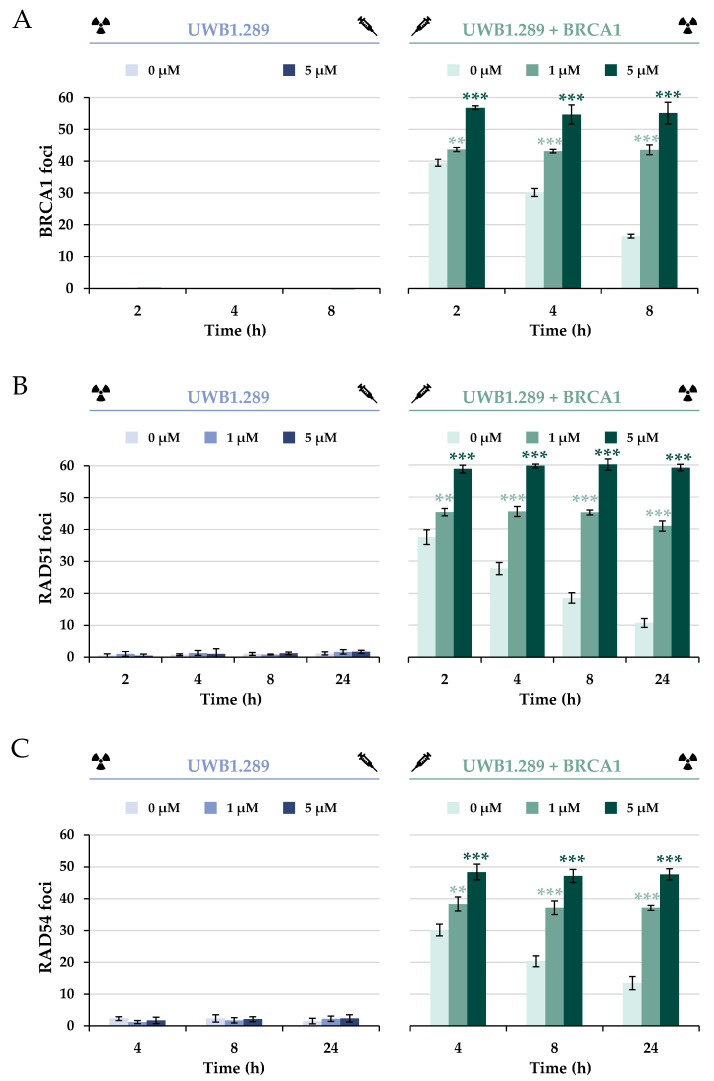
Immunostaining of BRCA1, RAD51, and RAD54 in UWB1.289 and UWB1.289 + BRCA1 cells after treatment with NaAsO_2_. Cells were pre-incubated with NaAsO_2_ for 18 h, irradiated with 1 Gy, and post-incubated with NaAsO_2_ for 2 h to 24 h, as indicated in the respective figure. Cells were stained against BRCA1 (**A**), RAD51 (**B**), or RAD54 (**C**), respectively, and manually counted. For each time point and treatment, foci in 40 cells of the G_2_ phase were counted, and the foci counts of the non-irradiated samples were subtracted from those of the irradiated batch. Shown are mean values ± standard deviations derived from three independent experiments. Statistically significant difference from control as determined using ANOVA followed by Dunnett’s T post hoc test: ** *p* ≤ 0.01, *** *p* ≤ 0.001.

To further investigate the impact of BRCA1 deficiency on specific repair proteins of HR upon IR, the protein BRCA1 itself was next analyzed (Figure 7A and Figure S6). The data confirm that the *BRCA1* mutation within exon 11 in the UWB1.289 cell line resulted in BRCA1 deficiency. Thus, no recruitment of BRCA1 to the IR-induced DSB in the G_2_ phase was detected in any of the treatments used or at any of the time points examined. In contrast, stably transfected UWB1.289 + BRCA1 cells showed significant expression of the protein as well as its binding to IR-mediated DSBs in G_2_. In addition, a decrease in BRCA1 foci was observed, suggesting effective repair of IR-induced DSBs.

Similarly to the foci formation of 53BP1, pre-incubation with 5 µM arsenite induced a concentration-dependent accumulation of BRCA1, providing further evidence for an increase in DNA DSBs and thus decreased repair. However, in contrast to the results obtained for the 53BP1 protein, arsenite completely inhibited the dissociation of BRCA1, starting already at the lowest concentration of 1 µM.

To test whether the lack of dissociation of the protein would affect the further repair process of HR, the downstream protein RAD51 was first examined (Figure 7B and Figure S7). The marker protein RAD51 forms a nucleoprotein filament on ssDNA and is essential for finding and invading homologous DNA sequences [20]. The next step was to investigate whether downstream recruitment of RAD54 would be affected (Figure 7C). RAD54 interacts with RAD51; it acts as a molecular motor during the homology search, stabilizes Rad51 filaments, and mediates RAD51 dissociation [21]. The results show that binding of RAD51 as well as RAD54 to IR-induced strand breaks was not possible in the BRCA1-deficient cells at any of the time points examined. In contrast, there was a clear association of RAD51 and RAD54 in the BRCA1-proficient cells in the G_2_ phase. Similarly to BRCA1, a distinct decrease in the binding of both proteins was observed over time, indicating efficient repair. However, as with BRCA1 foci, arsenite provoked an accumulation of RAD51 and RAD54 foci at all time points, indicating a completely impaired dissociation of both proteins.

**Figure 8:**
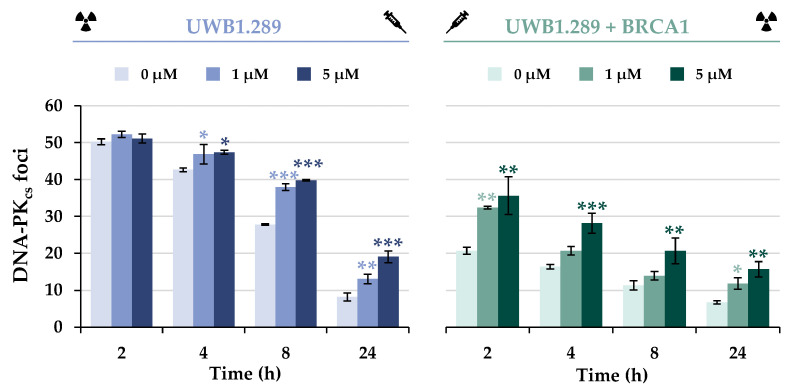
Immunostaining of DNA-PKcs in UWB1.289 and UWB1.289 + BRCA1 cells after treatment with NaAsO_2_. Cells were pre-incubated with NaAsO_2_ for 18 h, irradiated with 1 Gy, and post-incubated with NaAsO_2_ for 2 h to 24 h. Cells were stained against DNA-PKcs and manually counted. For each time point and treatment, foci in 40 cells of the G_2_ phase were counted, and the foci counts of the non-irradiated samples were subtracted from those of the irradiated batch. Shown are mean values ± standard deviations derived from three independent experiments. Statistically significant difference from control as determined using ANOVA followed by Dunnett’s T post hoc test: * *p* ≤ 0.05; ** *p* ≤ 0.01; *** *p* ≤ 0.001.

To elucidate the effect of BRCA1 deficiency and/or arsenite treatment on the alternative repair pathway NHEJ, DNA-PKcs foci formation was next examined (Figure 8 and Figure S8). In contrast to the HR proteins investigated so far, the NHEJ-associated protein DNA-PKcs was found to bind to the IR-induced DSB in both cell lines, but the number of foci was less than half in BRCA1-proficient cells compared to UWB1.289 cells. Nevertheless, progressive repair of DSBs by this pathway was evident in both cell lines, with a decreasing number of foci over 8 h repair time.

Co-treatment of UWB1.289 cells with arsenite did not result in additive or synergistic effects of IR-induced foci formation at the early repair times of 2 or 4 h. However, at later repair times, an increased number of foci was observed in arsenite-treated UWB1.289 cells compared to irradiation alone, suggesting slower repair in the presence of arsenite. In contrast, UWB1.289 + BRCA1 cells showed an increased number of foci after arsenite treatment compared to irradiation alone as early as 2 h, but the repair kinetics did not appear to be affected.

**Figure 9:**
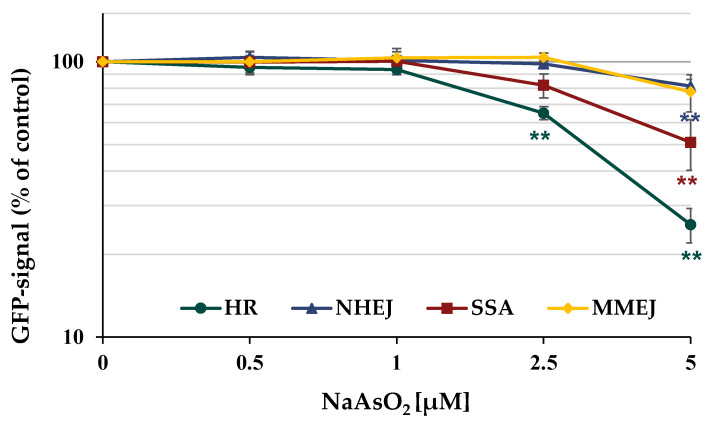
Impact of NaAsO_2_ on the repair of DSB measured by U2OS reporter assay. U2OS cells were transfected with ISce-I for 6 h. After removing the transfection cocktail, the cells were incubated with NaAsO_2_ for 66 h. Cells were subsequently trypsinized and harvested, and GFP-active cells were quantified via flow cytometry. The results are normalized to the transfected control. Shown are the mean values of three independent experiments performed in double determination ± SD. For each treatment, 50,000 events were counted. Statistical analysis between arsenite treatment and corresponding controls (** *p* ≤ 0.01) was performed using one-way ANOVA with post hoc Dunnett T.

Furthermore, the different repair pathways HR, NHEJ, SSA, and MMEJ were analyzed using a DSB repair reporter assay. U2OS cells carrying specific, inactive GFP expression cassettes, interrupted by an ISce-I restriction site were transfected with ISce-I to induce DNA DSBs. After the successful repair of these DNA DSBs by a specific repair pathway, the expression of active GFP can be quantified using flow cytometry. To obtain maximum GFP signals, cells were incubated for 66 h after transfection. The number of GFP-positive cells was quantified in the absence and presence of arsenite. In general, the results obtained by immunofluorescence were confirmed by the reporter assay shown in Figure 9. The number of GFP-positive cells for error-free HR decreased to about 65% of the control at concentrations as low as 2.5 µM NaAsO_2_ and to about 25% at 5 µM. While error-prone NHEJ and MMEJ were only slightly affected at the highest concentration, a reduction to approximately 50% of the untreated control was observed for SSA at 5 µM arsenite.

### 2.6. Cell Death

**Figure 10:**
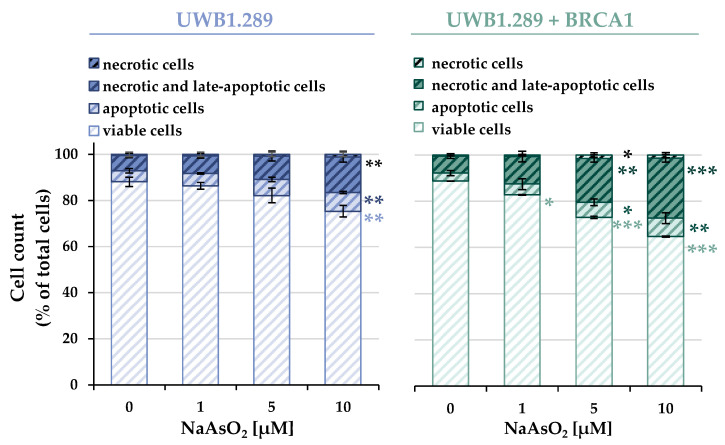
Analysis of apoptotic and necrotic cells after treatment with NaAsO_2_ for 26 h. To distinguish between necrotic, necrotic, late-apoptotic, apoptotic, and viable cells, cells were stained with Annexin V-FITC and propidium iodide (PI). Cell count was then analyzed using flow cytometry. Shown are mean values ± standard deviations derived from three independent experiments. Statistically significant difference from control as determined using ANOVA followed by Dunnett’s T post hoc test: * *p* ≤ 0.05; ** *p* ≤ 0.01; *** *p* ≤ 0.001.

As described above, treatment with NaAsO_2_ affected the expression of genes associated with apoptosis. To verify the results obtained at the functional level, cell death mechanisms in UWB1.289 and UWB1.289 + BRCA1 cells were subsequently analyzed using flow cytometry. The distribution of viable, necrotic, and apoptotic cells of the non-irradiated samples of both cell lines is shown in Figure 10, while the corresponding results of the irradiated cells are shown in Figure S9.

A concentration-dependent increase in apoptotic as well as necrotic and late apoptotic cells was observed in both BRCA1-deficient and -proficient cells. Similar to previous results, irradiation alone did not induce apoptotic or necrotic effects. Also, consistent with previous analyses, no additive or synergistic effects were observed with co-exposure compared to arsenite treatment alone.

While the basally distributed cellular fractions showed an almost identical distribution between the two cell lines, the concentration-dependent induction of cell death by arsenite was more pronounced in the BRCA1-proficient cells compared to the deficient cells. Incubation with 10 µM NaAsO_2_ resulted in a depletion of viable cells to 75% in BRCA1-deficient cells and 65% in BRCA1-proficient cells. The decrease in viability was mainly due to an increase in necrotic and late apoptotic cells, and also in apoptotic cells.

## 3. Discussion

In the present study, we aimed to elucidate the role of BRCA1 under basal conditions without additional DNA damage, under conditions of low-level DNA damage induced by 1 Gy IR, and after DNA damage induced by arsenite, with and without additional low-level IR. By comparing BRCA1-deficient and -proficient cells, it was confirmed that BRCA1 is essential for HR; its absence induces a shift towards more error-prone NHEJ. In BRCA1-proficient cells, arsenite reduced BRCA1 function by interfering with its dissociation from the sites of DNA damage, also resulting in a shift from HR to NHEJ.

BRCA1 plays an essential role in several mechanisms of the DDR. In addition to its involvement in error-free HR, multiple functions of other cellular signaling pathways of the DDR have been described, including cell cycle regulation, transcriptional regulation, protein ubiquitination, apoptosis, and chromatin remodeling [2].

Although arsenite has been shown to interfere with zinc finger proteins, little is known about arsenite-mediated interference with RING finger proteins, except for the binding of arsenite to the RING domains of an E3 ubiquitin ligase described in the introduction [15]. Since BRCA1 also harbors a Zn(II)-complexing RING finger structure, this tumor suppressor may also represent a potential target. Therefore, the present study aimed to evaluate the effect of arsenite on BRCA1 function by comparing BRCA1-deficient (UWB1.289) and BRCA1-proficient (UWB1.289 + BRCA1) cells. Concentrations from 1 µM to 10 µM were applied; the cytotoxicity of arsenite was comparable in both cell lines, and no significant effect on IR-induced cytotoxicity was observed. Interestingly, a pronounced intracellular accumulation of arsenic was found in both cell lines and thus independent of BRCA1 status.

To determine the effect of BRCA1 deficiency at the transcriptional level and thus possible differences in the gene expression profiles of UWB1.289 and UWB1.289 + BRCA1 cells, gene expression analyses were performed using high-throughput RT-qPCR. There was an increased basal *BRCA1* expression level in UWB1.289 + BRCA1 cells, whereas there was no BRCA1 expression in the deficient cells, confirming the respective BRCA1 status in both cell lines, due to the constitutive CMV (cytomegalovirus) promoter present in the pcDNA3 plasmid vector [22].

Regarding the effect of arsenite on the transcriptional DDR, a comparison of the gene expression profiles revealed a similar gene expression pattern in both cell lines, except for the DNA damage response (see below).

The most pronounced changes were observed in the oxidative stress response cluster upon treatment with arsenite, where changes in the target genes of the KEAP1-NRF2 pathway were detected. The highest induction was observed for *HMOX1*, encoding heme oxygenase 1, as a sensitive indicator of oxidative stress response; remarkably, a pronounced effect was already observed at the lowest concentration of arsenite, namely 1 µM. Other sensor genes for oxidative stress were also activated, although less pronounced, at 5 µM arsenite and higher, such as *HSPA1A.* Furthermore, small but dose-dependent inductions were observed for *G6PD*, *GCLC*, and *GSR* as well as for *NQO1* and *TXNRD1,* also target genes of the transcription factor NRF2, which regulate cellular GSH (glutathione) and TXN (thioredoxin) levels within the cellular antioxidant response. In contrast, slight repression was observed for the genes *NFKB1* and *NFKBIA*, whose expression is mediated by the redox-sensitive transcription factor NF-κB.

The increased expression levels of *MT1X* and *MT2A* upon NaAsO_2_ exposure suggest an involvement of the zinc-binding metal regulatory transcription factor MTF-1, which is activated by the release of zinc from metallothionein (MT) after metal excess or oxidative stress [23]. This also explains the up-regulation of *SLC30A1*, which encodes a proton-coupled zinc antiporter that mediates zinc efflux from cells to prevent toxicity [24].

Regarding the transcriptional DNA damage response, the induction of the DNA damage signaling genes *GADD45A* and *DDIT3* indicates arsenite-induced DNA damage. Interestingly, their activation was slightly more pronounced in BRCA1-deficient cells, suggesting some protection by BRCA1. In contrast, genes encoding specific DNA repair factors involved in basically all major DNA pathways were rather down-regulated, supporting previous reports where the expression of selected DNA repair genes was suppressed [25,26]. Interestingly, for the first time, the most pronounced repression was observed for *RRM2B* (ribonucleotide reductase regulatory TP53 inducible subunit M2B), which is required for DNA synthesis.

Finally, considerable changes were observed in cell cycle- and apoptosis-related genes. In particular, *CDKN1A*, which encodes the p53-dependent cell cycle regulator p21, was strongly up-regulated, whereas *E2F1* was slightly down-regulated. The slight down-regulation of *BCL2L1* and slight up-regulation of *PMAIP1*, *TNFRSF10B*, and *VEGFA* also suggested modulation of apoptotic pathways by arsenite.

To further characterize the effect of arsenite on cell cycle progression and control as well as on apoptosis, flow cytometric studies were performed focusing on the comparison between BRCA1-deficient and BRCA1-proficient cells. Comparison of the basal cell cycle distribution revealed an increased population of BRCA1-proficient cells in the G_1_ phase, supporting a direct involvement of BRCA1 in the regulation of the G_1_/S checkpoint. Thus, BRCA1 has been shown to both directly and indirectly affect the phosphorylation of RB and consequently prevent the release of the transcription factor E2F [27,28]. Treatment with arsenite resulted in a G_2_/M arrest in the BRCA1-proficient, but not in the BRCA1-deficient cells, supporting a direct role of BRCA1 in this checkpoint as well [29]. A dose-dependent increase in the G_2_/M cell population after arsenite treatment was previously reported in BRCA1-proficient and p53-deficient cell lines [30]. Using microscopic analysis, the authors demonstrated mainly an increase in mitotic cells, without undergoing a cell cycle arrest in the G_2_ phase. They further postulated that the M-arrest is associated with increased induction of apoptosis [30]. It should be noted that the flow cytometric studies performed in this study did not distinguish between G_2_ and M arrest. Nevertheless, to the best of our knowledge, the present study was the first to demonstrate the direct involvement of BRCA1 in the functional control of cell cycle regulation after arsenite exposure by comparing BRCA1-proficient and -deficient cells.

The expression changes in apoptosis-related genes described above and the repression of the *NFKB1-* and *NFKBIA*-activated genes may affect arsenite-induced cell death by modulating the transcriptional activity of NF-κB, possibly triggering the induction of apoptosis. Therefore, the results of the gene expression analyses were followed up using flow cytometry. NaAsO_2_ induced cell death in both BRCA1-deficient and -proficient cells in a concentration-dependent manner. However, UWB1.289 + BRCA1 cells showed slightly enhanced cell death compared to UWB1.289 cells, suggesting a functional role of BRCA1 in these mechanisms. This observation is consistent with the proposed role of BRCA1 in chemotherapy. Thus, Quinn and colleagues postulated that BRCA1 acts as a differential modulator of chemotherapy-induced apoptosis depending on the type of cellular damage. A pronounced sensitizing effect and thus increased apoptosis was observed in the case of spindle poisons, whereas decreased apoptosis occurred in response to DSB-inducing agents such as etoposide [31].

As described above, the induction of the sensor gene *GADD45A* and the increased expression of *DDIT3* were indicative of DNA damage, whereas the expression of most other genes in this cluster, except BRCA1, was slightly decreased by NaAsO_2_ in a concentration-dependent manner. Taken together, the gene expression results support the general conclusion that arsenite causes impairment of various repair mechanisms in addition to the induction of DNA damage. In the next steps of the present study, we focused on the impact of BRCA1 and arsenite on DNA DSB repair, specifically on NHEJ and HR. To this end, IF-staining was used to analyze the binding of various DNA repair proteins in the presence or absence of arsenite, comparing BRCA1-deficient and BRCA1-proficient cells. As a first step, DSB damage recognition was investigated by selecting the 53BP1 protein. According to the present results, BRCA1 deficiency did not seem to affect the detection of DSBs, but rather increased 53BP1 binding, indicating a higher number of DSB; thus, BRCA1 seems to promote the repair of DSBs. Treatment with NaAsO_2_ had no adverse effect on the binding of this protein. However, arsenite induced a higher number of foci both immediately after irradiation and after 8 h of repair, indicating an increased number of unrepaired DSBs, but no effect on 53BP1 protein dissociation.

It is well known that NHEJ is active in all phases of the cell cycle, whereas HR is only available in the late S and G_2_ phases. In the G_2_ phase, the earlier repair steps are carried out by the fast NHEJ, which switches to the much slower but more precise HR [17]. Therefore, the results may provide preliminary evidence that the increased number of 53BP1 foci, observed after incubation with 5 μM arsenite in the BRCA1-deficient cells at the later time point of 8 h, may be due to an impaired HR. On this basis, the effect of BRCA1 deficiency and arsenite exposure on the recruitment of specific repair proteins of HR (BRCA1, RAD51, and RAD54) was further investigated.

As expected, UWB1.289 cells showed no association of BRCA1 protein after either irradiation alone or additional NaAsO_2_ treatment, confirming a functional BRCA1 deficiency. The downstream proteins Rad51 and Rad54 were also not recruited to DNA damage sites in BRCA1-deficient cells. Based on the known co-localization of DNA repair proteins to nuclear foci, it could be hypothesized that the lack of BRCA1 association also impairs the recruitment of other proteins and thus the mechanism of error-free HR.

In contrast, irradiation of the stably transfected UWB1.289 + BRCA1 cells and subsequent analysis of the binding and dissociation kinetics of BRCA1, RAD51, and RAD54 suggested successful restoration of HR repair capacity in this cell line.

The recruitment of BRCA1 to UWB1.289 + BRCA1 cells was also observed in the presence of NaAsO_2_. However, in contrast to control cells, arsenite completely inhibited the dissociation of BRCA1 from damage sites in a concentration-dependent manner. This may be due to an interaction of arsenite with the RING finger domain of BRCA1. Up to now, arsenite has been known to interfere with zinc-binding structures such as the zinc finger of PARP1 [32] or XPA [10] and the RING finger of RNF20 or RNF40 [15].

To test whether the lack of dissociation of the protein might negatively affect the further repair process of HR, the downstream proteins RAD51 and RAD54 were next examined. Although their association was not affected by arsenite, their dissociation was disturbed, similar to the results with BRCA1 foci formation. Again, this could be due to direct impairment of the repair proteins due to their thiol-rich amino acid composition. While RAD54 is a potential target for trivalent arsenic because of its zinc finger, RAD51 also is a target because of its critical thiol groups. Arsenite binding to thiol groups and their oxidation can adversely affect the function of DNA repair proteins [12,13]. The observed impaired dissociation ability of BRCA1, RAD51, and RAD54 could, therefore, be due to a change in their secondary structure and thus their function. Regarding the binding of BRCA1 and RAD51, our results seem to contradict those published previously by Zhang and coworkers [15], who observed decreased binding of BRCA1 and RAD51 in HeLa cells after laser-induced DNA DSB induction. This may be due to differences in cell lines and DNA DSB induction in the Zhang study, versus the IR-induced DNA DSB used in the present study, as well as higher arsenite concentrations (5 µM and 20 µM) examined at one time point, namely 10 min after DSB induction. Overall, this is the first study to examine the time course of association and dissociation of the respective repair factors in the presence of arsenite.

It is conceivable that the previously observed impaired HR induced by BRCA1 deficiency and NaAsO_2_ treatment led to a shift in repair mechanisms, thereby promoting NHEJ. Similar results have been reported in previous studies in which a reduced level of repair by HR and a slightly increased incidence of NHEJ repair were observed in BRCA1-deficient mouse embryonic stem cells [33,34].

To test this hypothesis, the NHEJ-associated protein DNA-PKcs was next examined. It was shown that both association and dissociation of DNA-PKcs and thus NHEJ were not inhibited by BRCA1 deficiency, but rather activated compared to UWB1.289 + BRCA1 cells. Thus, the shift from error-free HR to error-prone NHEJ after IR-induced damage by BRCA1 deficiency was also demonstrated at the level of specific DNA repair proteins in the present study.

Similar considerations can also be postulated for arsenite treatment. Possibly, the increased number of DNA-PKcs foci in BRCA1-proficient cells after exposure to NaAsO_2_ is due to a reduced HR repair capacity and thus enhanced NHEJ; the dissociation of this protein was not affected. Data from the GFP-based DSB repair reporter assays also confirmed that arsenite reduced the capacity for largely error-free HR, whereas error-prone NHEJ was only slightly impaired. The latter observation is consistent with the results of Zhang and coworkers, who described reduced DSB repair via the HR and NHEJ pathways after arsenite treatment [15]. Overall, arsenite was shown to induce a shift in DSB repair mechanisms from error-free HR to error-prone NHEJ after IR-induced damage.

## 4. Materials and Methods

### 4.1. Cell Culture, Irradiation, and Drug Treatment

UWB1.289 cells (ATCC CRL-2945) were grown as monolayers in 50% Mammary Epithelial Cell Growth Medium (MEGM) and 50% Roswell Park Memorial Institute (RPMI) medium containing 3% FBS, 100 U/mL penicillin, and 100 µg/mL streptomycin. UWB1.289 + BRCA1 cells (ARCC CRL-2946), the same cell line transfected with wild-type BRCA1, were grown in the same medium with G418 supplementation (200 µg/mL). Both cell lines were cultured at 37 °C with 5% CO_2_ in the air and 100% humidification. Gamma-irradiation was performed with a Faxitron CellRad System (Faxitron Bioptics LLC, Tucson, AZ, USA). Logarithmically growing cells were pretreated with NaAsO_2_, solved in cell culture medium for 18 h, irradiated afterward in the presence of NaAsO_2,_ or left unirradiated and post-incubated for up to 24 h.

U2OS cells were grown in DMEM medium containing 10% fetal bovine serum, 100 U/mL penicillin, and 100 µg/mL streptomycin at 37 °C in 5% CO_2_. Gamma-irradiation was performed with a Faxitron CellRad System (Faxitron Biotics LLC, Tucson, AZ, USA).

### 4.2. Cytotoxicity Assays

For cell number, 3 × 10^5^ UWB1.289 and 2.5 × 10^5^ UWB1.289 + BCRA1 cells were seeded. Logarithmically growing cells were treated with NaAsO_2_ and irradiated as indicated in the drug treatment section, washed with PBS, trypsinized, and collected in a fresh medium. The cell number was determined using a CASY^®^ Cell Counter (OLS OMNI Life Science GmbH & Co KG, Bremen, Germany).

For analyzing ATP content, CellTiter-Glo^®^ Luminescent Cell Viability Assay Kit (Promega GmbH, Walldorf, Germany) was applied. Cell cultivation and incubation were performed in 96-well plates. Briefly, 1 × 10^4^ UWB1.289 cells and 0.75 × 10^4^ UWB1.289 + BRCA1 cells per well were seeded. Logarithmically growing cells were treated with NaAsO_2_ and irradiated as indicated in the drug treatment section. After removing the incubation medium, the cells were washed twice with PBS and 100 µL of fresh medium was added to the wells. CellTiter-Glo^®^ Luminescent Cell Viability Assay was carried out following the manufacturer’s protocol. The plate was equilibrated for 30 min in the dark at room temperature, and 100 µL of CellTiter-Glo^®^ reagent was added. Chemiluminescence was measured on the Infinite^®^ 200 Pro microplate reader (Tecan Group Ltd., Männedorf, Switzerland) after short-term orbital shaking and a further 10 min to stabilize the signal. The ATP content and, therefore, the reduction in cell viability was expressed as a percentage normalized to non-treated control cells.

### 4.3. Atomic Absorption Spectroscopy (AAS)

For determination of the cellular arsenic status, 1 × 10^6^ UWB1.289 and 0.8 × 10^6^ UWB1.289 + BRCA1 cells were seeded. Logarithmically growing cells were treated with NaAsO_2_ for 18 h, washed with PBS, trypsinized, and collected in ice-cold PBS containing 10% FBS. The cells were washed twice in PBS and counted via CASY^®^ Cell Counter for cell number and mean cell volume. The cells were incubated with the ashing mixture of 65% HNO_3_/30% H_2_O_2_ (1/1) for 1 h, evaporated at 95 °C, and resuspended in water. The arsenic content was then determined by GF-AAS (Perkin Elmer Atomic Absorption Spectrometer PinAAcle 900T) using AAS elemental standard solutions. The cellular arsenic concentration in µM was calculated using the following equation, with *c*(*AAS*) as arsenic concentration determined by GF-AAS in µg/L, *M*(*As*) as the molecular mass of arsenic in g/mol, *CC* as the cell count of the respective cell suspension and *CV* as the cell volume in L^−1^, determined using a CASY^®^ Cell Counter.

$$c\;\left(cellular\;arsenic\right)=\frac{c\;(AAS)}{M\;\left(As\right)*CC*CV}$$

### 4.4. High-Throughput RT-qPCR

For gene expression analysis, 4 × 10^5^ UWB1.289 and UWB1.289 + BRCA1 cells were seeded. Logarithmically growing cells were treated with NaAsO_2_ and irradiated as indicated in the drug treatment section, washed with PBS, trypsinized, resuspended in ice-cold PBS containing 10% FBS, and collected by centrifugation. Afterward, the RNA was isolated by applying the MN NucleoSpin^®^ RNA Plus KIT (Machinerey-Nagel, Dueren, Germany) according to the manufacturer’s instructions. The following high-throughput RT-qPCR was performed with Fluidigm dynamic arrays on the BioMark™ system, as described previously [16]. Data were evaluated using Fluidigm Real-Time PCR Analysis as well as GenEx software, version 5.3.6.170. Normalization was performed using five reference genes (*ACTB*, *B2M*, *GAPDH*, *GUSB*, and *HPRT1*). Changes in transcription levels of the target genes were presented as a log_2_-fold change compared to untreated controls by calculating relative quantities according to the ΔΔCq method [35].

### 4.5. Immunofluorescence (IF)

For immunofluorescence analysis, 8 × 10^4^ UWB1.289 and UWB1.289 + BRCA1 cells were seeded on coverslips. Logarithmically growing cells were treated with NaAsO_2_ and irradiated as indicated in the drug treatment section. Afterward, the cells were fixed with 3.7% formaldehyde/PBS for 10 min at room temperature at the indicated time points. Permeabilization was carried out with 0.2% Triton X-100/PBS for 5 min at 4 °C. Subsequently, the non-specific binding sites were blocked overnight at 4 °C by using 2% BSA/PBS. The samples were incubated with primary anti-CENP-F antibody (Abcam ab5 (rabbit); Invitrogen MA1-23185 (mouse)) in combination with primary anti-53BP1 antibody (Santa Cruz sc-515841 (E-10)), anti-DNA-PKcs Phospho (Thr2609) antibody (Biolegend 612902), anti-BRCA1 antibody (Abcam ab16780), anti-Rad51 antibody (Abcam ab63801) or anti-Rad54 antibody (Santa Cruz sc-374598 (F-11)) for one hour at room temperature or overnight at 4 °C. All antibodies were diluted in 2% BSA/PBS. After washing with PBS, cells were incubated with Alexa Fluor^®^ 488-conjugated anti-mouse antibody (Invitrogen A-11001) and Cy3-conjugated anti-rabbit antibody (Jackson 111-165-003) for one hour at room temperature or overnight at 4 °C. Afterward, the cells were counterstained by mounting with Vectashield Mounting Medium with DAPI (Vector Laboratories Inc., Burlingame, CA, USA) and analyzed using immunofluorescence microscopy (Zeiss, Jena, Germany).

### 4.6. Analysis of Apoptosis and Cell Cycle Distribution via Flow Cytometry

For apoptosis and cell cycle analysis, 3.5 × 10^5^ UWB1.289 and UWB1.289 + BRCA1 cells were seeded. Logarithmically growing cells were treated with NaAsO_2_ and irradiated as indicated in the drug treatment section. Subsequently, the media and the trypsinized cells were transferred into a 15 mL tube. After collecting by centrifugation, the supernatant was discarded, and the cells were resuspended in PBS and divided for the determination of apoptosis and the analysis of cell cycle distribution.

For cell cycle analysis, cells were fixed with ice-cold 96% ethanol and stored overnight at −20 °C. Afterward, the fixed cells were centrifuged, the supernatant was discarded and the cells were washed with PBS, followed by centrifugation. Cells were resuspended in DAPI staining solution (Partec, Münster, Germany) and incubated on ice for 30 min in the dark. Fluorescence was measured using a BD LSRFortessa flow cytometer (BD, Heidelberg, Germany) with a violet laser of 405 nm excitation and a bandpass filter of 450/50 nm. To determine the cell cycle distribution, the fluorescence signal was plotted against the count in a histogram.

For the determination of apoptosis, the cell suspension was centrifuged, and the supernatant was discarded. The cells were resuspended in a mix of 200 µL Ringer solution (146 nM NaCl, 402 nM KCl, 297 nM CaCl_2_) + 0.25 µL propidium iodide (PI) (62.5 ng/mL) + 1 µL annexin V-FITC (450 ng/mL) and incubated on ice for 30 min in the dark. While PI was used to determine necrotic cells, annexin V-FITC stains apoptotic cells. Fluorescence was measured using a BD LSRFortessa flow cytometer (BD, Heidelberg, Germany) with a blue laser of 488 nm excitation and a bandpass filter of 530/30 nm for the FITC signal, and a bandpass filter of 695/40 nm for the PI signal. Since the absorption spectra of FITC and PI overlapped, compensation was performed before analysis. To distinguish between vital, apoptotic, necrotic, and late apoptotic and necrotic populations, the FITC signal was plotted against the PI signal in a dot plot.

### 4.7. U2OS Reporter Assay

The U2OS reporter assay includes a panel of four U2OS osteosarcoma cell lines, each for one DNA DSB repair mechanism, established originally by Gunn and Stark [36,37]. In this assay, U2OS cells were subjected to transfection with an individual, inactive GFP expression cassette, containing a restriction site for the rare-cutting endonuclease ISce-I. Subsequently, DNA DSBs were induced using the ISce-I endonuclease. The transfection with ISce-I was carried out for 6 h using the Effectene Transfection Reagent (Qiagen, Venlo, The Netherlands). To determine transfection efficiency and serve as a toxicity control, some U2OS cells were also co-transfected with pEGFP-N1.

Following the removal of the transfection cocktail, the cells were exposed to NaAsO_2_ for the duration of the experiment. After 72 h post-transfection, the U2OS cells were harvested by trypsinization, and resuspended in PBS, and the number of GFP-positive cells was quantified using flow cytometry (Becton Dickinson LSR Fortessa FACS, Heidelberg, Germany), as described previously [38]. A total of 50,000 events were collected for analysis.

## 5. Conclusions

In the present study, the role of BRCA1 was investigated under basal conditions after low-level DNA damage induced by 1 Gy IR and after arsenite treatment with and without co-treatment with IR. With the exception of *BRCA1* gene expression, there were almost no differences between BRCA1-proficient and -deficient cells on the transcriptional level in the absence of additional treatment. Similarly, the transcriptional DDR was not activated in either cell line upon treatment with low dose (1 Gy) IR. In contrast, arsenite induced distinct transcriptional responses, including a pronounced DNA damage response. The picture changed significantly at the functional level: Even in the absence of additional DNA damage, the cell cycle distribution differed in BRCA1-proficient and -deficient cells, and no cell cycle control was evident after arsenite treatment in the absence of BRCA1. Regarding the association of specific DNA repair factors at sites of IR-induced DNA damage, no proteins involved in HR were recruited in the absence of BRCA1, but a pronounced shift towards NHEJ occurred. In the case of arsenite treatment, this shift was also evident in BRCA1-proficient cells. Although BRCA1 was still able to bind to the damage, its dissociation was completely inhibited, and the same was true for RAD51 and RAD54, which are also involved in HR, suggesting that HR can be initiated, but not completed in the presence of arsenite. This was also confirmed in a DSB repair reporter assay, where arsenite strongly inhibited HR but had only a little effect on NHEJ. In contrast, no effect was observed on the association and dissociation of DNA-PKcs, suggesting a shift from the largely error-free HR to the more error-prone NHEJ. Taken together, arsenite appears to affect BRCA1 function and thus genomic stability, possibly by inducing structural changes within its RING finger domain.

## Data Availability

The data presented in this study are available upon request from the corresponding author (A.H.) for the researchers of academic institutes who meet the criteria to access the confidential data.

## Supplementary Materials

The following supporting information can be downloaded at: https://www.mdpi.com/article/10.3390/ijms241814395/s1.

## Abbreviations

AAS	atomic absorption spectroscopy
BRCA1	breast cancer type 1 susceptibility protein
CENP-F	centromere protein F
DDR	DNA damage response
DSB	double-strand break
DSBR	double-strand break repair
GSH	glutathione
HR	homologous recombination
IARC	International Agency for Research on Cancer
IF	immunofluorescence;
IR	ionizing radiation
MEGM	Mammary Epithelial Cell Growth Medium
MT	metallothionein
MTF-1	metal-regulatory transcription factor 1
NHEJ	non-homologous end-joining
PI	propidium iodide
ROS	reactive oxygen species
RPMI	Roswell Park Memorial Institute
TXN	thioredoxin
