# Supplementary material for: Arsenite Impairs BRCA1-Dependent DNA Double-Strand Break Repair, a Mechanism Potentially Contributing to Genomic Instability
Source: Int J Mol Sci. 2023 Sep 21;24(18):14395. doi: 10.3390/ijms241814395 (PMC10532266; doi:10.3390/ijms241814395)
Supplement: Supplementary file 1 [file ijms-24-14395-s001.zip › ijms-2577487-supplementary.pdf]

Supplementary information to:

Arsenite impairs BRCA1-dependent DNA double-strand break repair, a mechanism potentially contributing to genomic instability

Tizia Matthäus [1], Sandra Stößer [1], Hatice Yasemin Seren [1], Vivien M. M. Haberland[1] and Andrea Hartwig [1*]

1 Department of Food Chemistry and Toxicology, Institute of Applied Biosciences (IAB), Karlsruhe Institute of Technology (KIT), Adenauerring 20a, 76131 Karlsruhe, Germany

* Correspondence: andrea.hartwig@kit.edu (A.H.)

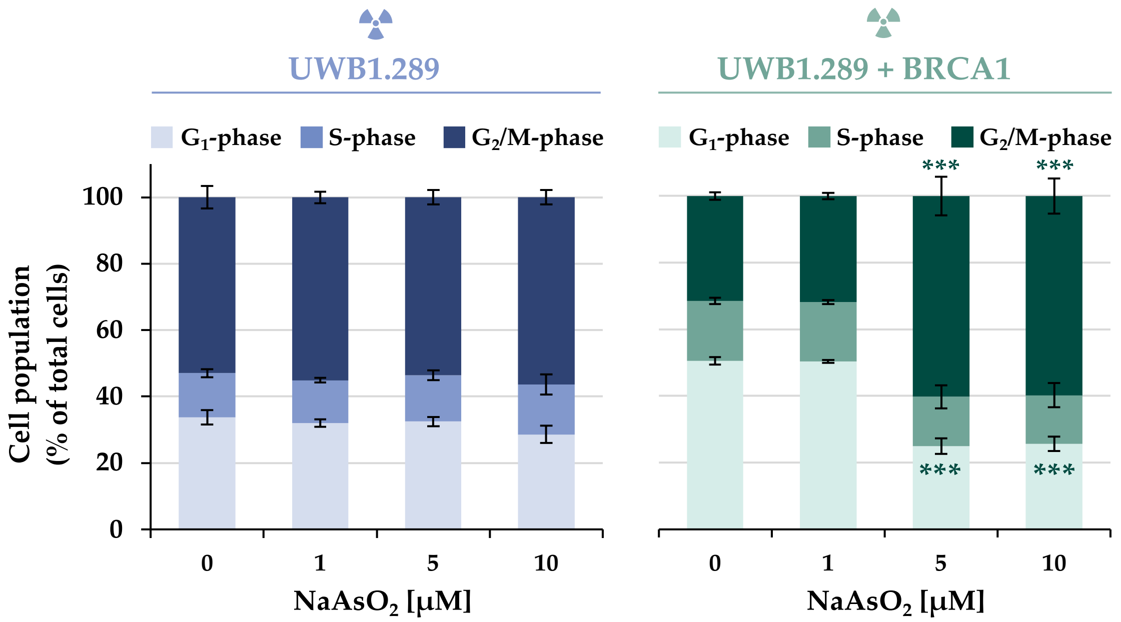

Figure S1: Cell cycle distribution of UWB1.289 and UWB1.289 + BRCA1 cells after treatment with $NaAsO_2$. Cells were pre-incubated with $NaAsO_2$ for 18 h, irradiated with 1 Gy, and post-incubated with $NaAsO_2$ for 8 h. Cell cycle distribution was analyzed by DAPI staining using flow cytometry. Shown are mean values ± standard deviations derived from three independent experiments. Statistically significant difference from control as determined by ANOVA followed by Dunnett`s T post hoc test: ***$p \leq 0.001$.

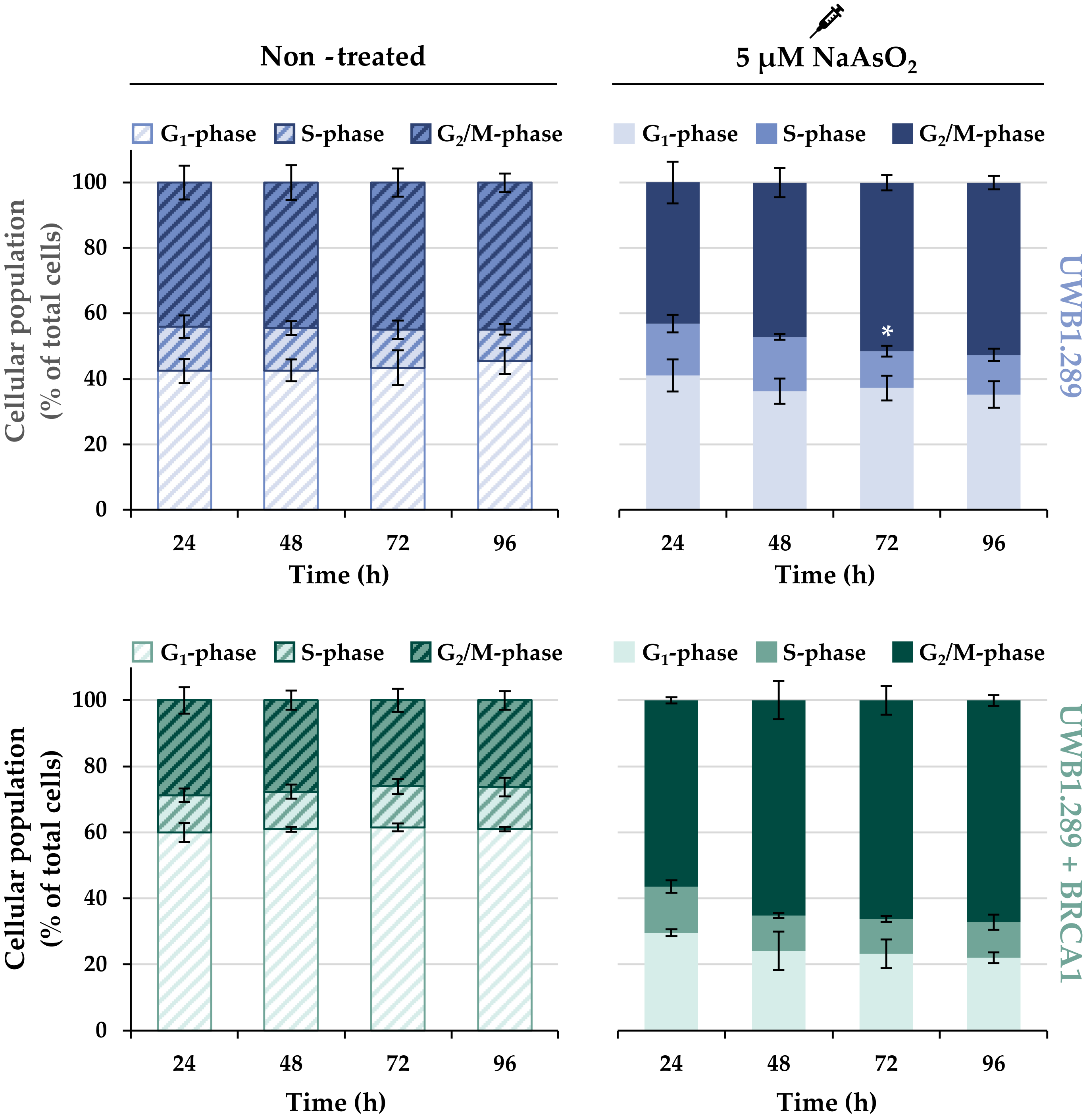

Figure S2: Time-dependent analysis of the cell cycle distribution of UWB1.289 and UWB1.289 + BRCA1 cells after treatment with $NaAsO_2$. Cells were treated with 5 µM $NaAsO_2$ and post-incubated for 24 h to 96 h. Cell cycle distribution was analyzed by DAPI staining using flow cytometry. Shown are mean values ± standard deviations derived from three independent experiments. Statistically significant difference from control as determined by ANOVA followed by Dunnett`s T post hoc test: *$p \leq 0.05$.

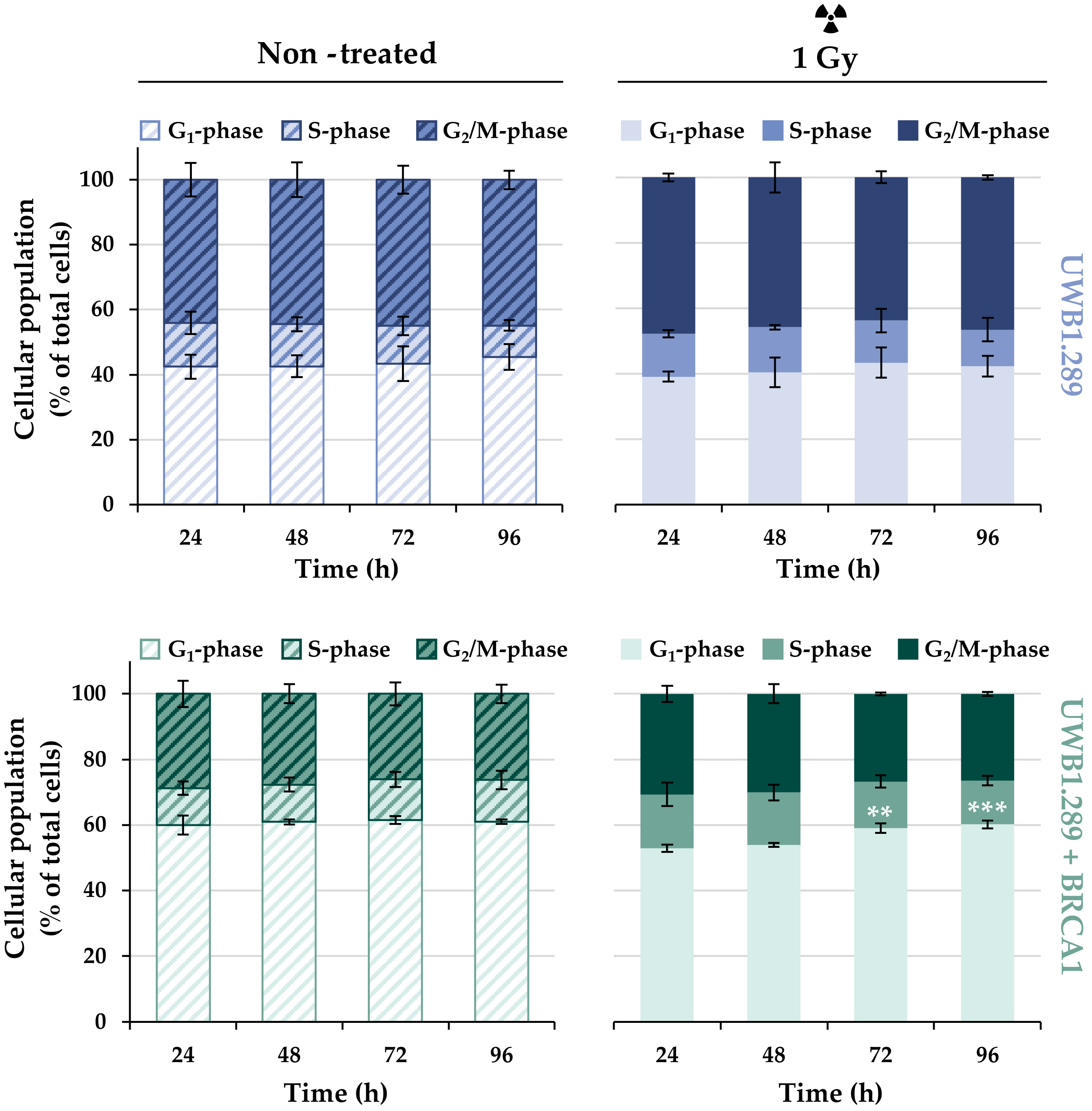

Figure S3: Time-dependent analysis of the cell cycle distribution of UWB1.289 and UWB1.289 + BRCA1 cells after exposure to IR. Cells were irradiated with 1 Gy and post-incubated for 24 h to 96 h. Cell cycle distribution was analyzed by DAPI staining using flow cytometry. Shown are mean values ± standard deviations derived from three independent experiments. Statistically significant difference from control as determined by ANOVA followed by Dunnett`s T post hoc test: $^{**}p \leq 0.01$, $^{***}p \leq 0.001$.

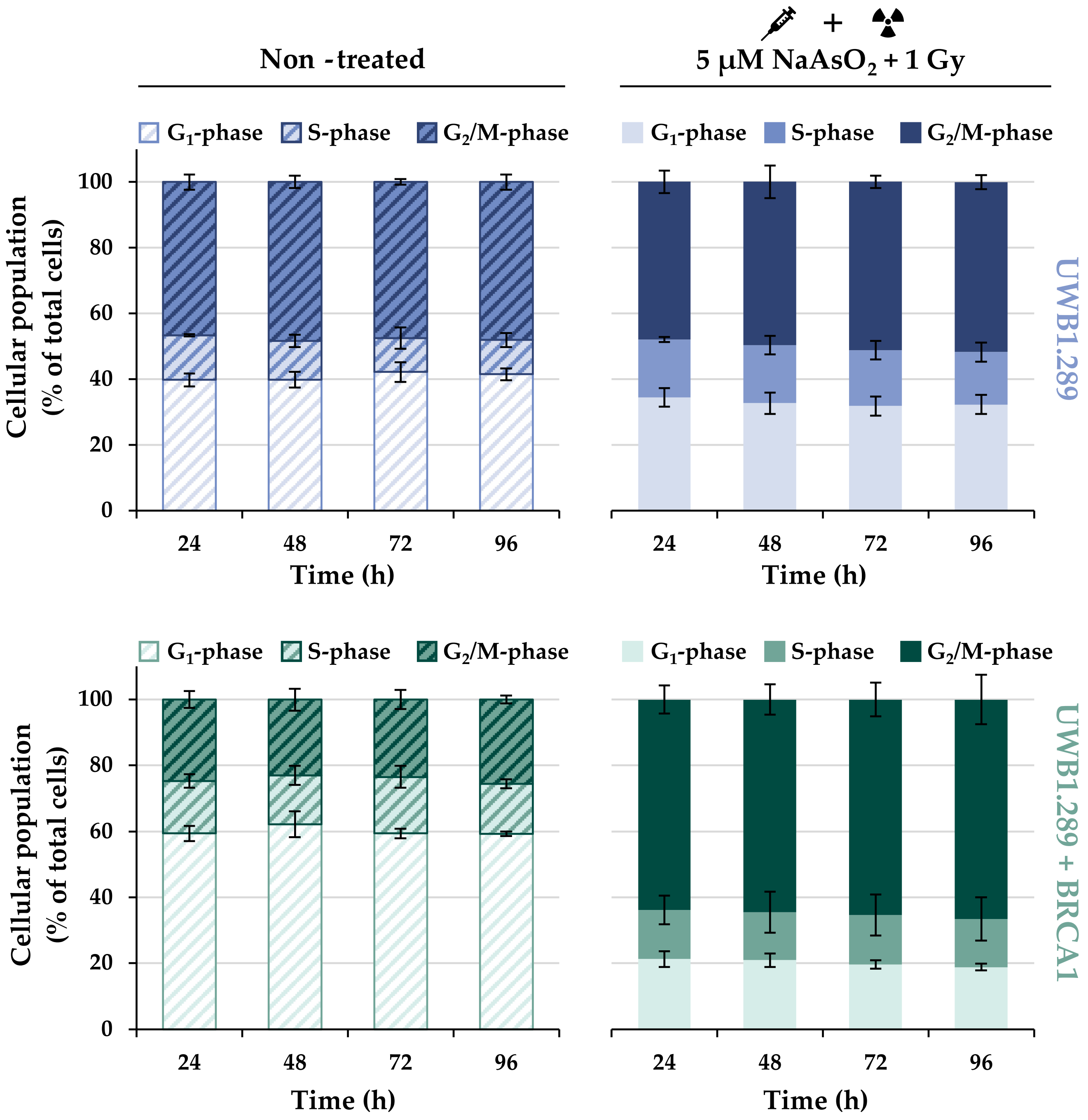

Figure S4: Time-dependent analysis of the cell cycle distribution of UWB1.289 and UWB1.289 + BRCA1 cells after co-exposure to $NaAsO_2$ and IR. Cells were pre-incubated with $NaAsO_2$ for 18 h, irradiated with 1 Gy, and post-incubated with $NaAsO_2$ for 24 h to 96 h. Cell cycle distribution was analyzed by DAPI staining using flow cytometry. Shown are mean values ± standard deviations derived from three independent experiments. Statistically significant difference from control as determined by ANOVA followed by Dunnett`s T post hoc test.

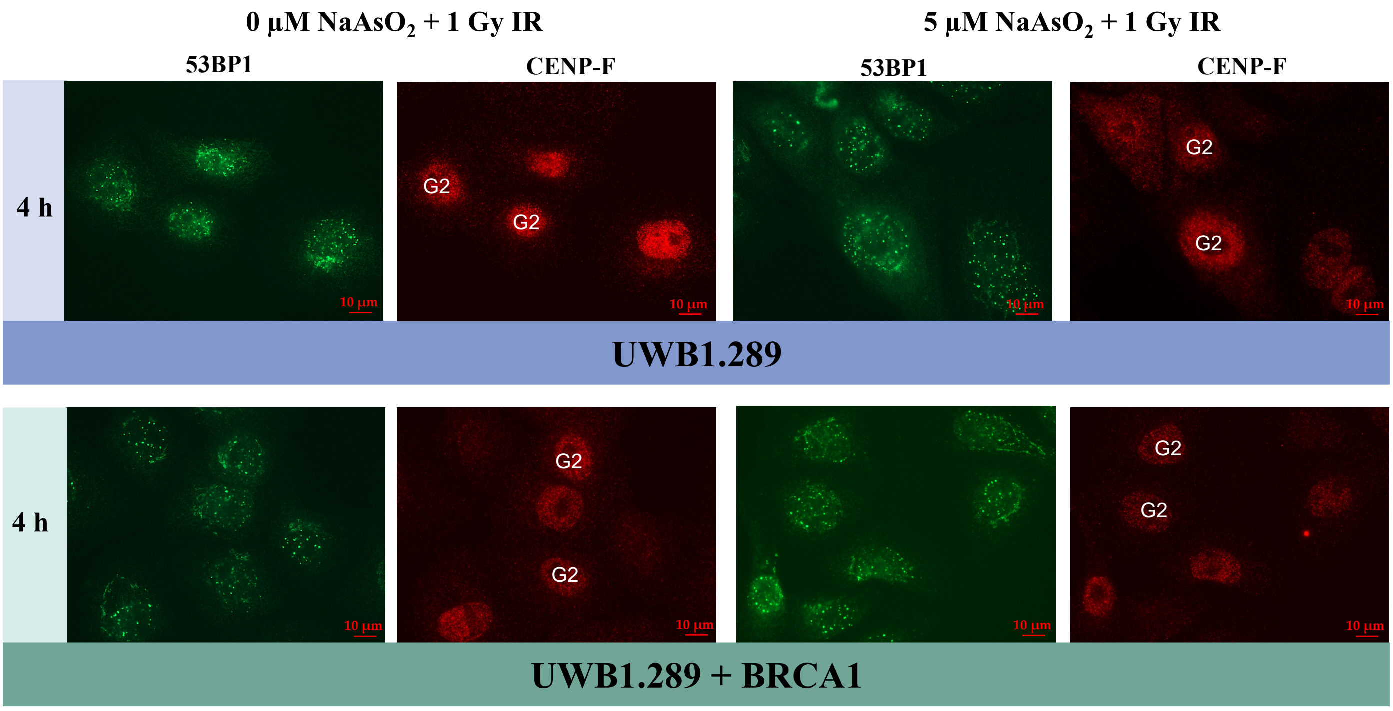

Figure S5: Impact on 53BP1 foci formation in UWB1.289 and UWB1.289 + BRCA1 cells after treatment with $NaAsO_2$. Cells were pre-incubated with $NaAsO_2$ for 18 h, irradiated with 1 Gy, and post-incubated with $NaAsO_2$ 4 h. The cells were stained using specific primary and secondary antibodies against 53BP1, as well as CENP-F against the centromere protein F.

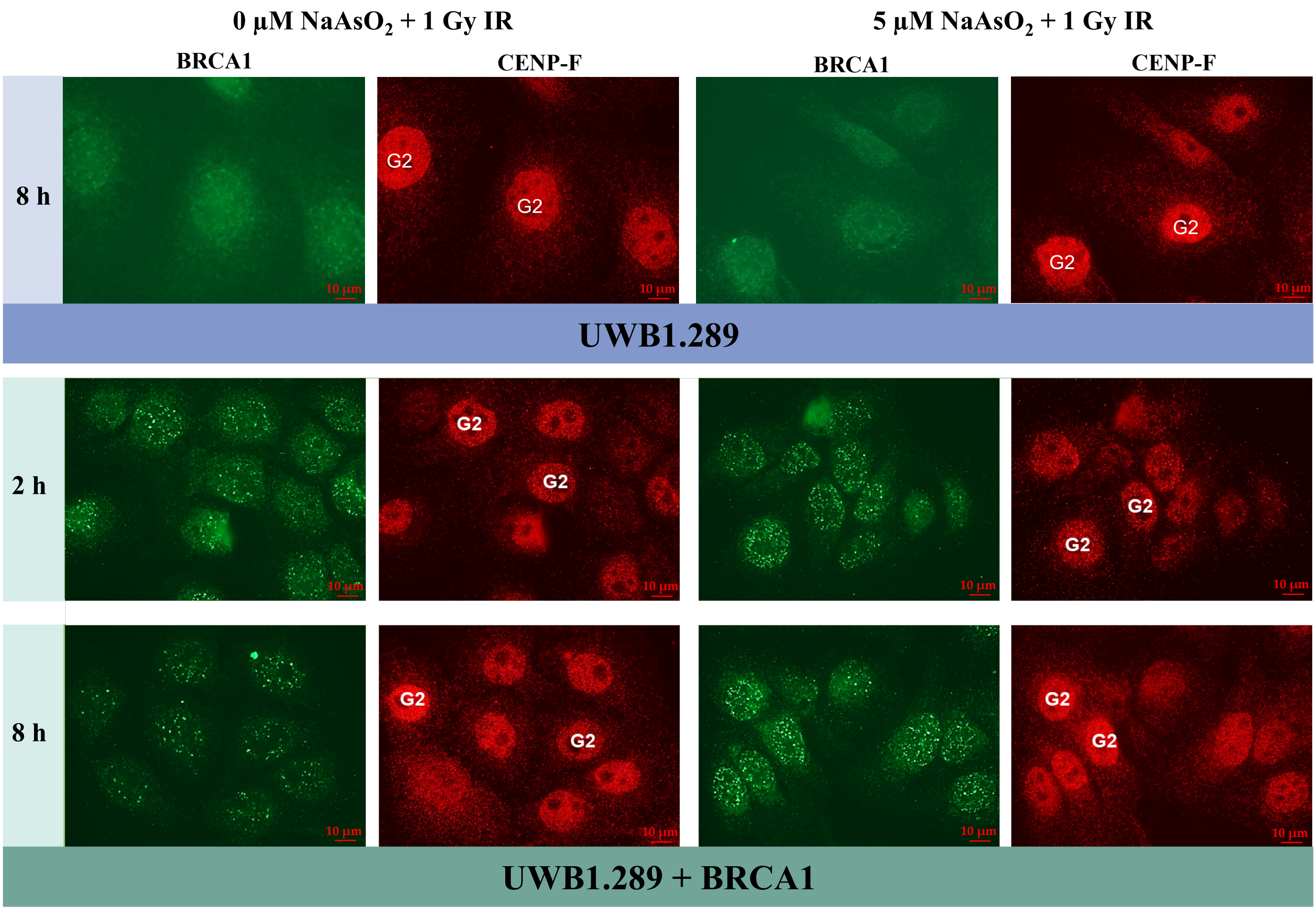

Figure S6: Impact on BRCA1 foci formation in UWB1.289 and UWB1.289 + BRCA1 cells after treatment with $NaAsO_2$. Cells were pre-incubated with $NaAsO_2$ for 18 h, irradiated with 1 Gy, and post-incubated with $NaAsO_2$ 2 h and 8 h. The cells were stained using specific primary and secondary antibodies against 53BP1, as well as CENP-F against the centromere protein F.

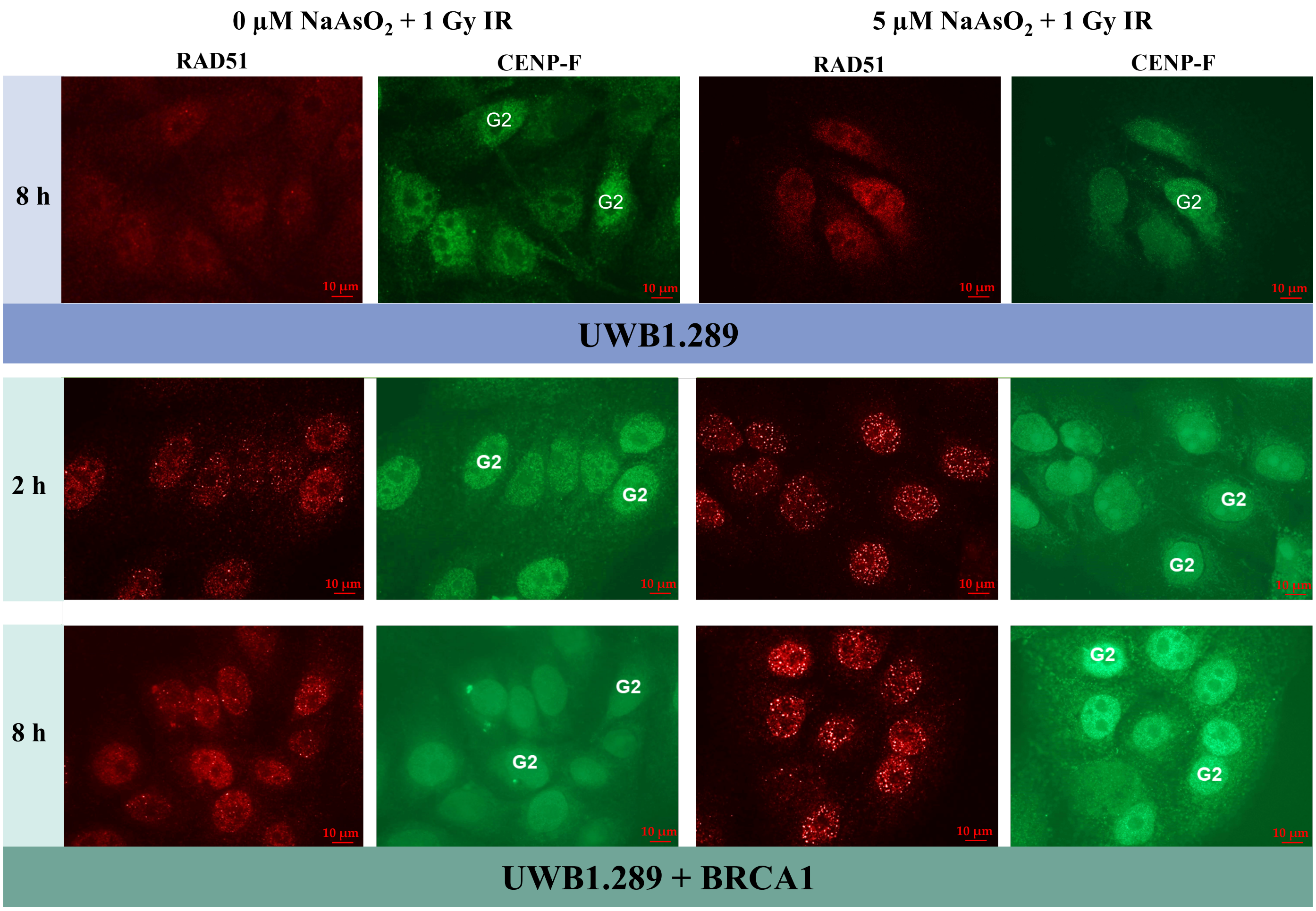

Figure S7: Impact on RAD51 foci formation in UWB1.289 and UWB1.289 + BRCA1 cells after treatment with $NaAsO_2$. Cells were pre-incubated with $NaAsO_2$ for 18 h, irradiated with 1 Gy, and post-incubated with $NaAsO_2$ 2 h and 8 h. The cells were stained using specific primary and secondary antibodies against 53BP1, as well as CENP-F against the centromere protein F.

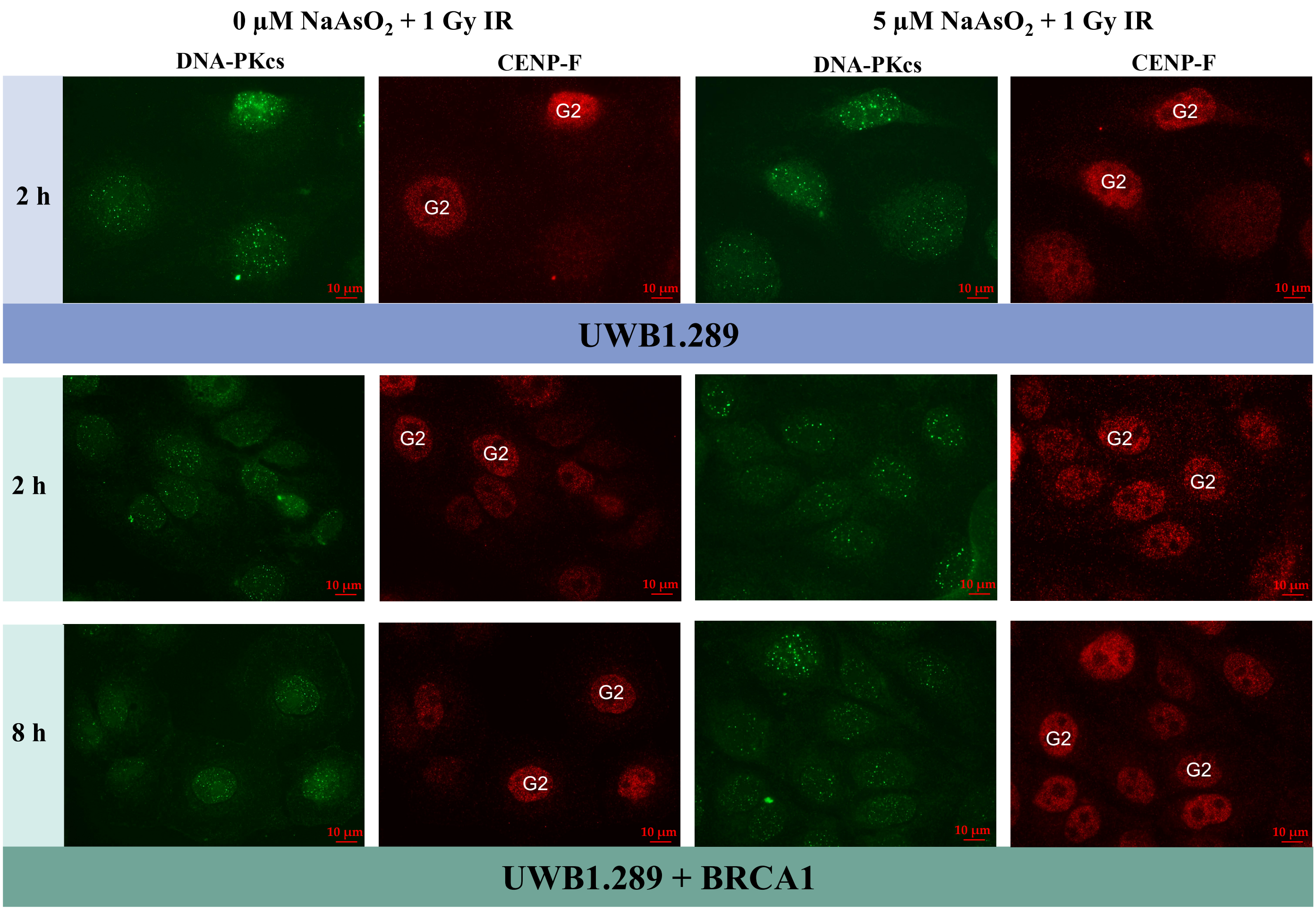

Figure S8: Impact on DNA-PKcs foci formation in UWB1.289 and UWB1.289 + BRCA1 cells after treatment with $NaAsO_2$. Cells were pre-incubated with $NaAsO_2$ for 18 h, irradiated with 1 Gy, and post-incubated with $NaAsO_2$ 2 h and 8 h. The cells were stained using specific primary and secondary antibodies against DNA-PKcs, as well as CENP-F against the centromere protein F.

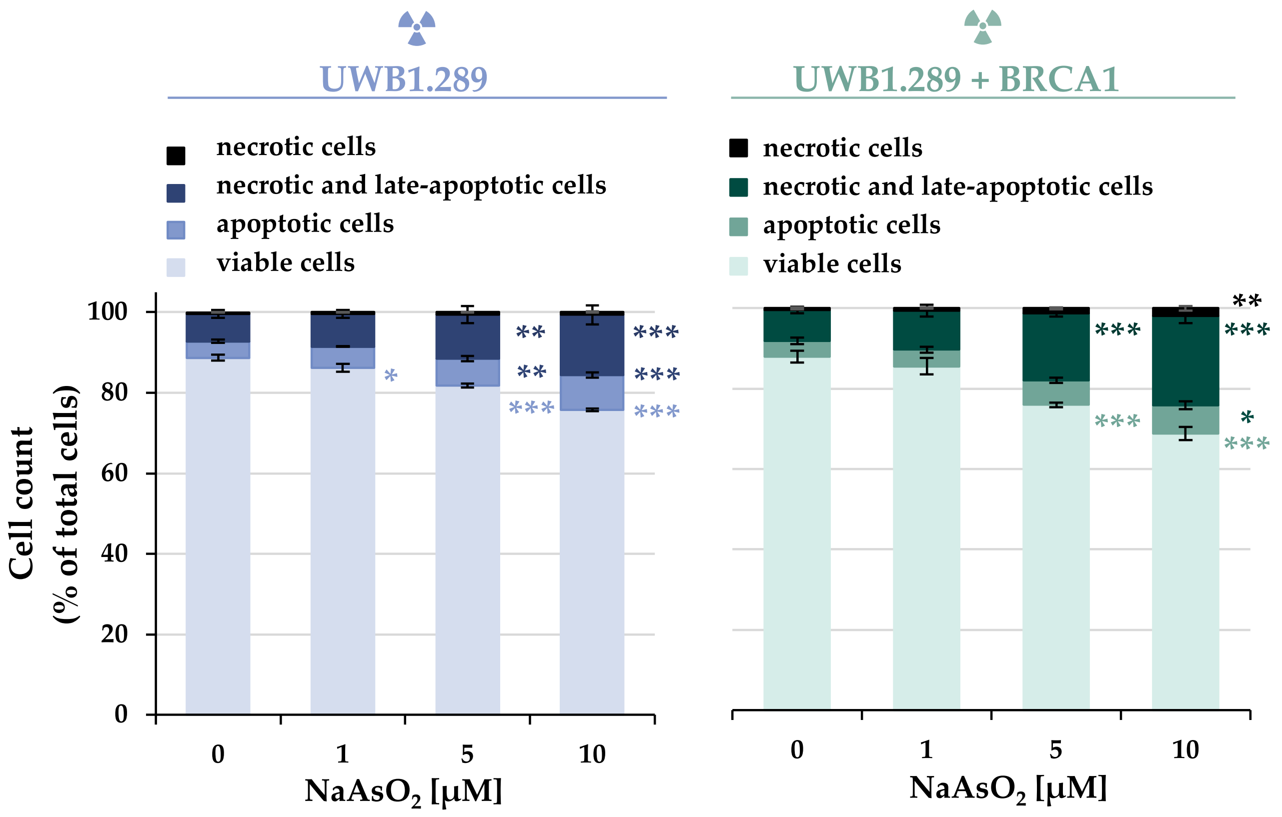

Figure S9: Analysis of apoptotic and necrotic cells after treatment with $NaAsO_2$. Cells were pre-incubated with $NaAsO_2$ for 18 h, irradiated with 1 Gy, and post-incubated with $NaAsO_2$ for 8 h To distinguish between necrotic, necrotic, and late-apoptotic, apoptotic, and viable cells, cells were stained with Annexin V-FITC and propidium iodide (PI). Cell count was then analyzed using flow cytometry. Shown are mean values ± standard deviations derived from three independent experiments. Statistically significant difference from control as determined by ANOVA followed by Dunnett`s T post hoc test: $^*p \leq 0.05$, $^{**}p \leq 0.01$, $^{***}p \leq 0.001$.
